# Suppression of DC-SIGN and gH Reveals Complex, Subset-Specific Mechanisms for KSHV Entry in Primary B Lymphocytes

**DOI:** 10.3390/v13081512

**Published:** 2021-07-31

**Authors:** Nancy Palmerin, Farizeh Aalam, Romina Nabiee, Murali Muniraju, Javier Gordon Ogembo, Jennifer Totonchy

**Affiliations:** 1 Biomedical and Pharmaceutical Sciences Department, Chapman University School of Pharmacy, Chapman University, Irvine, CA 92618, USA; palmerin@chapman.edu (N.P.); aalam@chapman.edu (F.A.); nabie100@mail.chapman.edu (R.N.); 2 Department of Immuno-Oncology, Beckman Research Institute of City of Hope, Duarte, CA 91010, USA; mmuniraju@coh.org (M.M.); jogembo@coh.org (J.G.O.)

**Keywords:** KSHV, glycoprotein H, lymphotropism, DC-SIGN, viral entry

## Abstract

Kaposi sarcoma-associated herpesvirus (KSHV) is the causative agent of multiple cancers in immunocompromised patients including two lymphoproliferative disorders associated with KSHV infection of B lymphocytes. Despite many years of research into the pathogenesis of KSHV associated diseases, basic questions related to KSHV molecular virology remain unresolved. One such unresolved question is the cellular receptors and viral glycoproteins needed for KSHV entry into primary B lymphocytes. In this study, we assess the contributions of KSHV glycoprotein H (gH) and the cellular receptor DC-SIGN to KSHV infection in tonsil-derived B lymphocytes. Our results show that (1) neither KSHV-gH nor DC-SIGN are essential for entry into any B cell subset, (2) DC-SIGN does play a role in KSHV entry into tonsil-derived B cells, but in all B cell subtypes alternative entry mechanisms exist, (3) KSHV-gH can participate in KSHV entry into centrocytes via a DC-SIGN independent entry mechanism, and (4) in the absence of KSHV-gH, DC-SIGN is required for KSHV entry into centrocytes. Our results provide a first glimpse into the complexity of KSHV entry in the lymphocyte compartment and highlight that multiple subset-dependent entry mechanisms are employed by KSHV which depend upon multiple cellular receptors and multiple KSHV glycoproteins.

## 1. Introduction

Kaposi Sarcoma Herpesvirus (KSHV) is an oncogenic herpesvirus and one of only two human herpesviruses known to infect B lymphocytes. KSHV lymphotropism results in two lymphoproliferative disorders, PEL and MCD [1,2]. Despite decades of research related to KSHV pathogenesis, essential questions remain unanswered related to how KSHV invades a new human host and particularly how the virus invades the human immune system. Studies addressing these gaps are particularly critical to inform the rational development of prophylactic vaccine strategies to limit KSHV transmission. 

The highly conserved glycoproteins gB, gH and gL form the canonical core entry machinery for all herpesviruses, and various additional glycoproteins, which vary between herpesviruses, work together with these core proteins to mediate the broad cellular tropism characteristic of human herpesviruses [3]. There is a considerable body of literature characterizing the cellular receptors and KSHV glycoproteins involved in viral entry in vitro. However, no study to date has succeeded in completely abrogating KSHV infection via inhibition of a specific cellular receptor, suggesting that KSHV universally employs redundant entry mechanisms involving multiple cellular receptors and glycoproteins [4]. 

In epithelial, endothelial and fibroblast cell types, KSHV infection is facilitated by attachment of gB, gH/gL and/or K8.1 to heparin sulfate proteoglycans, however our recent work showed that HSPG play no role in infection of primary tonsil-derived B lymphocytes [5]. Similarly, a previous study from our group recently showed that KSHV-gH is essential for entry into all adherent cell types tested, but is not required for entry into the MC116 lymphoma cell line [6]. Thus, there is accumulating evidence that KSHV uses different mechanisms for entry into B cells compared to adherent cells.

DC-SIGN, also known as, Dendritic Cell-Specific ICAM-3-Grabbing Non-integrin, CD209, is a type II C-type (calcium-dependent) lectin receptor [7] and is highly expressed in cells of the immune system such as dendritic cells (DCs), in dermal and mucosal tissues, monocytes, macrophages, B lymphocytes from peripheral blood, and tonsillar B lymphocytes [8,9]. DC-SIGN plays a critical role in the innate and adaptive immune response by facilitating immune cell signaling, adhesion and migration [10]. DC-SIGN is utilized as an entry receptor by many viruses such as Ebola virus, HIV-1, Hepatitis C, KSHV, SARS coronavirus, and Dengue virus [11]. Studies have demonstrated that KSHV uses DC-SIGN as an entry receptor in dendritic cells and macrophages (Rappocciolo, 2006 #1195), and neutralization of DC-SIGN was previously shown to inhibit KSHV infection of activated blood-derived and resting tonsil-derived B lymphocytes [8]. KSHV-gB binds to DC-SIGN in a dose dependent manner, but this interaction has yet to be deemed essential for KSHV entry in any cell type [12]. 

In this study, our primary objectives are to evaluate the role of KSHV-gH in entry into primary B lymphocytes, and determine whether DC-SIGN is the primary receptor for KSHV in B lymphocytes as has been previously reported [8]. Finally, we seek to examine whether either factor (DC-SIGN as a receptor and KSHV-gH as a glycoprotein) dictates the susceptibility of any specific B cell subsets to KSHV infection. Our results demonstrate that both DC-SIGN and gH are dispensable for infection of tonsil-derived B lymphocytes and suppression of these factors does not substantially alter KSHV tropism in B lymphocytes. Interestingly, by working with these two factors in combination, we are able to establish that KSHV has two mechanisms for entry into the centrocyte subset: one dependent on gH and the other dependent on DC-SIGN. Taken together, our results reveal that mechanisms of KSHV entry into B lymphocytes are complex, multi-factoral and subset-specific. 

## 2. Materials and Methods

### 2.1. Preparation of Cell-Free Recombinant KSHV Virions

iSLK cell lines harboring BAC16-KSHV-WT-eGFP [13] and BAC16-KSHV-∆gH-eGFP [6] were cultured in Dulbecco’s Modified Eagle Medium (DMEM) supplemented with 10% Cosmic Calf Serum (CCS), PSG, puromycin (1 µM), G418 (250 µg/mL), and hygromycin (1.2 mg/mL) at 37 °C in 5% CO_2_. For virus preparations, 12 × T185 flasks at 80–90% confluence were stimulated for 72 h with 3 mM sodium Butyrate and 2 µM doxycycline hyclate. At 3 days post induction, supernatants were clarified by centrifugation at 1700 rpm for 12 min at 4 °C and filtered with a 0.45 µm vacuum filter. Virions were pelleted out of clarified supernatant over 25% sucrose in TNE (50 mM Tris [pH 7.4], 100 mM NaCl, 0.1 mM EDTA, pH 7.4) by centrifugation at 22,000 rpm for 2 h. Virus pellets were resuspended in 2 mL TNE and stored at −80 °C. Infectious titer doses were determined for iSLK-BAC16-KSHV-WT-eGFP by serial dilution infection on human fibroblasts and quantified at 3 days post infection via flow cytometry. Infectious titer doses were determined for iSLK-BAC16-KSHV-∆gH-eGFP by calculating equal genome copy number to that of iSLK-BAC16-KSHV-WT-eGFP via quantitative PCR (qPCR).

### 2.2. Isolation of Primary Lymphocytes from Human Tonsil

De-identified human tonsil specimens were obtained from the National Disease Research Interchange (NDRI) following routine tonsillectomies. Less than 24 h post-surgery, tonsil specimens were shipped and delivered in DMEM+PSG to the laboratory. Primary lymphocytes were extracted via dissection and maceration of tonsil tissues in RPMI media. Lymphocyte-containing media were passed through a 40 µm filter, and pelleted at 1500 rpm for 5 min. Red blood cell lysing solution (0.15 M ammonium chloride, 10 mM potassium bicarbonate, 0.1 M EDTA) was utilized to lyse red blood cells present in the lymphocyte preparation. Following 3 min of RBC lysis, lymphocytes were diluted in 50 mL of PBS, manually counted and pelleted at 1500 rpm for 5 min. Aliquots of 1 × 10^8^ cells were resuspended in 1 mL of freezing media (90% FBS, 10% DMSO) and cryopreserved.

### 2.3. Isolation and KSHV Infection of Total B lymphocytes 

Tonsil primary lymphocyte suspensions were thawed at 37 °C, slowly diluted to 5 mL with RPMI, and pelleted at 1500 rpm for 5 min. Pellets were resuspended in 1 mL RPMI with 20% FBS, 100 µg/mL DNase I, and 100 µg/mL Primocin. Cells were maintained in a low-binding 24 well plate at 37 °C and 5% CO_2_ incubator for two hours. After recovery, total lymphocytes were counted and total B cells were isolated using Mojosort™ Human Pan B cell isolation kit (Biolegend 480082) according to manufacturer’s instructions. Bound cells that were non-B cells were retained and maintained in 1 mL RPMI, 20% FBS at 37 °C and 5% CO_2_ incubator.

Recombinant KSHV virion preparations were titrated on human fibroblasts and an ID20 was calculated by linear regression. The equivalent ID20 dose from fibroblasts (in µL/cell) was diluted in serum free RPMI media and used to infect 1 million B cells. For experiments including KSHV-∆gH, the WT virus was used at ID20 doses as described above, and KSHV-∆gH was used at an equivalent genome dose based on the WT stock. In all experiments, Mock-infected cultures were included as an internal reference for the GFP positive signal and to allow analysis of culture-specific effects. B cells in infection media were spinoculated at 1000 rpm for 30 min at 4 °C in 12 × 75 mm round bottom tubes. After spinoculation, tubes were incubated at 37 °C for an additional 30 min. After incubation, infected cultures were transferred to X-ray irradiated CDW32 L cells in a 48 well plate and reconstituted with 20% fetal bovine serum, 100 µg/mL of Primocin and 1 million cells from the bound fraction of the B cell isolation for a final concentration of 4 million cells/mL. Reconstituted lymphocyte cultures were incubated at 37 °C, 5% CO_2_ for the duration of the experiment. 

### 2.4. DC-SIGN Neutralization 

Total B lymphocytes were isolated as in 2.3 and were incubated with Human DC-SIGN/CD209 Antibody [anti-DC-SIGN mAb] (R&D systems MAB161-100) at varying concentrations (0 µg/mL, 2.5 µg/mL, 5 µg/mL) for 30 min on ice prior to infection as described above.

### 2.5. DC-SIGN Depletion 

Total B lymphocytes were isolated as described above, and were further separated into DC-SIGN+ (bound) and DC-SIGN- (unbound) fractions using CD209 (DC-SIGN) MicroBeads (Miltenyi 130-092-868) according to manufacturer’s instructions. Following separation, half of the DC-SIGN- B cells were reconstituted with DC-SIGN+ B cell fraction and the remaining cultures remained depleted. These depleted and reconstituted samples were Mock-infected or infected with KSHV-WT and KSHV-∆gH and cultured as described above.

### 2.6. Flow Cytometry Analysis

At day 0 (baseline) or at 3 days post-infection (3 dpi), 5 × 10^5^ lymphocytes were aliquoted into a 96-well round bottom plate and pelleted at 1500 rpm for 5 min. Resuspension of the pellet was performed with 100 µL PBS containing zombie violet fixable viability stain (Biolegend Cat# 423113) and incubated on ice for 15 min. After incubation, 100 µL PBS, containing 2% FBS and 0.5% BSA and 0.1% sodium azide (FACS Block) was added to the wells. Cells were pelleted at 1500 rpm for 5 min and resuspended in 200 µL FACS Block followed by a 10 min incubation on ice. Cells were pelleted at 1500 rpm for 5 min and resuspended in 50 µL of PBS containing 0.5% BSA and 0.1% sodium azide (FACS Wash), 10 µL BD Brilliant Stain Buffer Plus (BD 566385) and antibodies as follows: IgD-BUV395 (2.5 µL/test BD 563823), CD77-BV510 (2.0 µL/test BD 563630), CD138-BV650 (2 µL/test BD 555462), CD27-BV750 (2 µL/test BD 563328), CD19-PerCPCy5.5 (2.0 µL/test BD 561295), CD38-APC (10 µL/test BD 560158), CD20-APCH7 (2 µL/test BL 302313), and DC-SIGN- PE-Cy7 (2 µL/test BD 330114) and incubated on ice for 15 min. After incubation, 150 µL FACS Wash was added. Cells were pelleted at 1500 rpm for 5 min followed by two washes with FACS Wash. Cells were collected in 200 µL FACS Wash for flow cytometry analysis. Sample data and appropriate compensation controls were acquired on a BD Fortessa X20 flow cytometer and analyzed using FlowJo Software.

### 2.7. RT-PCR Analysis for Viral Transcripts 

At 3 days post infection, 2 × 10^6^ lymphocytes were harvested into Trizol and an equal volume of DNA/RNA shield (Zymo Research R110-250, Irvine, CA, USA) was added. RNA extraction was performed using Zymo Directzol Microprep (Zymo Research R2060) according to manufacturer instructions. RNA was eluted in 10 µL H_2_O containing 2U RNase inhibitors and a second DNase step was performed for 30 min using the Turbo DNA-Free kit (Invitrogen AM1907M, Waltham, MA, USA) according to manufacturer instructions. Nested RT-PCR amplicon for GAPDH was designed based on NCBI gene id 2597 and LANA and K8.1 assays were designed based on the BAC16 reference sequence (Genbank MK733609.1) One-step RT-PCR cDNA synthesis and preamplification of GAPDH, LANA and K8.1 transcripts was performed on 15 ng of total RNA using the Superscript III One-step RT-PCR kit (ThermoFisher 12574026, Waltham, MA, USA) and 2 µM outer primers for each target gene (Table 1). Duplicate no RT (NRT) control reactions were assembled for each sample containing only Platinum Taq DNA polymerase (Thermofisher 15966005) instead of the Superscript III RT/Taq DNA polymerase mix. After cDNA synthesis at 50 °C for 15 min 20 cycles of target pre-amplification was performed with an annealing temperature of 60 °C and 30second extension at 68 °C. Then, 2 µL of pre-amplified cDNA or NRT control reaction was used as template for multiplexed real-time PCR reactions using TaqProbe 5× qPCR MasterMix -Multiplex (ABM MasterMix-5PM), 5% DMSO, primers at 900 nM and probes at 250 nM against target genes (Table 1) and analyzed using a standard 40 cycle program on a Biorad real time thermocycler. Data is represented as quantitation cycle (Cq) and assays in which there was no detectable Cq value were set numerically as Cq = 41 for analysis and data visualization.

### 2.8. Statistical Analysis 

Data plots and statistical analysis were performed in Rstudio software (version 7.0) using reshape2 [14], and tidyverse [15] packages. Statistical analysis was performed using R package: rstatix [16]. Specific statistical tests and the resulting values are described in detail in the corresponding figure legends. 

## 3. Results

### 3.1. DC-SIGN Is Widely Expressed among Tonsil B Cell Subsets 

**Figure 1:**
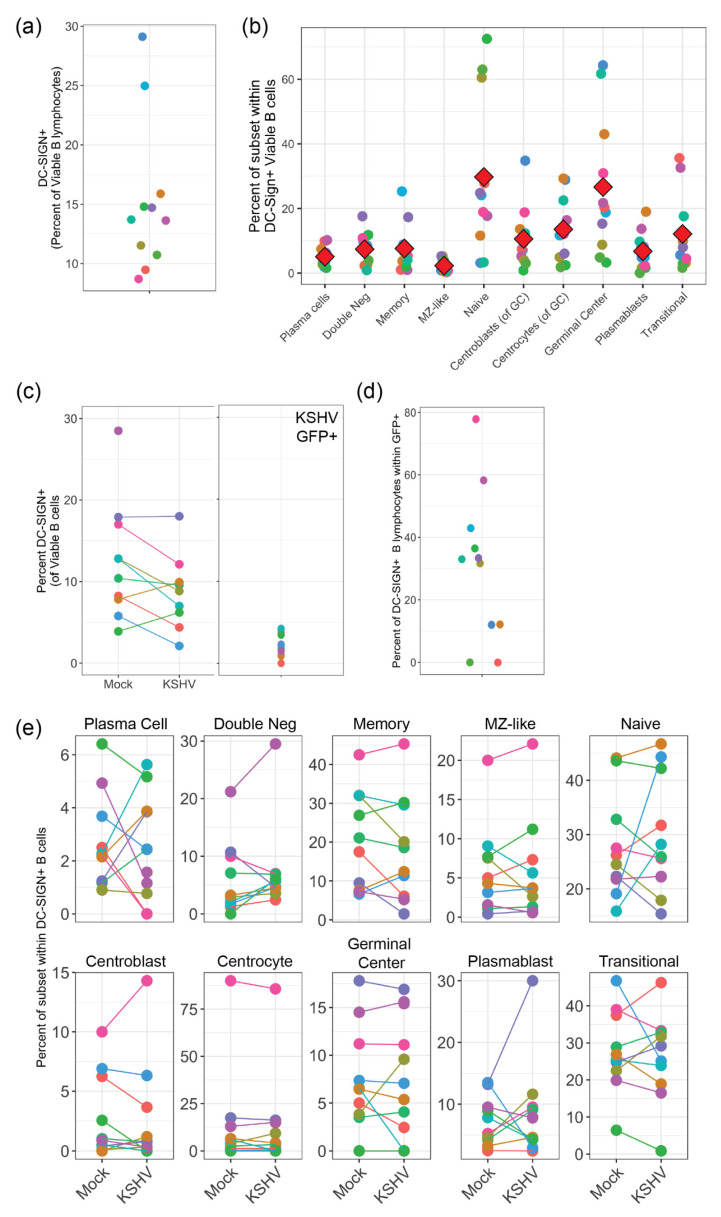
DC-Sign expression in human tonsil with and without KSHV infection. (**a**) Flow cytometry analysis of DC-SIGN expression on the surface of viable B cells in 11 unique tonsil lymphocyte specimens at day 0 (baseline). (**b**) Analysis as in (**a**) showing the distribution of B cell subsets within DC-SIGN+ B cells. Red diamonds indicate the mean of 11 tonsil samples. (**c**) DC-SIGN expression at 3 days post-infection (dpi) in Mock-infected and KSHV-infected cultures (left panel, n.s.) or in KSHV-infected GFP+ B cells (right panel). (**d**) Frequency of DC-SIGN expression on GFP+ B lymphocytes in KSHV-infected cultures at 3 dpi. (**e**) Distribution of B cell subsets within DC-SIGN+ B-cells as in (**b**) for Mock and KSHV-infected cultures at 3 dpi (n.s.). Where indicated, data point color scheme indicates unique tonsil specimens and can be compared between sub-panels within each portion of the figure.

We first wanted to establish the subset-specific expression of DC-SIGN in tonsil-derived B cells prior to infection. This analysis allows us to determine if DC-SIGN expression correlates with subset-specific susceptibility to KSHV infection and also provides a baseline for comparison of infected cultures to determine whether our lymphocyte culture system, or KSHV infection, changes DC-SIGN expression over time. These results reveal that most tonsil lymphocyte preparations contained 10–15% DC-SIGN+ B cells (Figure 1a). These results are slightly lower than those previously reported [17]. When we performed immunophenotypic B cell subset analysis (Table 2, Figure S1) on DC-SIGN positive B cells, we determined that most B cell subsets, with the exception of MZ-like, were present within the DC-SIGN+ population and germinal center, naive, and transitional subsets were highly represented (Figure 1b).

### 3.2. KSHV Infection Does Not Alter DC-SIGN Expression on B Cells and Expression of DC-SIGN on KSHV-Infected Cells Is Highly Variable

To determine whether our culture system and/or KSHV infection alters the distribution of DC-SIGN within B cell subsets, we performed KSHV infection and analysis at 3 dpi for 10 tonsil lymphocyte preparations quantitating DC-SIGN+ B cells and the distribution of B cell subsets within the DC-SIGN+ population for both Mock and KSHV-infected cultures. These results reveal that there was no statistically significant change in overall DC-SIGN expression on B lymphocytes in Mock vs. KSHV-infected cultures (Figure 1c, left panel). Moreover, we found that although significant numbers of DC-SIGN+ B cells were present in KSHV-infected cultures, few of these DC-SIGN+ cells were KSHV infected (as shown by the expression of the GFP reporter that is constitutively expressed from the recombinant KSHV genome), indicating that infection is not particularly enriched in the DC-SIGN+ fraction (Figure 1c, right panel). However, since overall KSHV infection is low in these cultures (~1% of B cells) we analyzed the data to examine the frequency of DC-SIGN expression on KSHV-infected (GFP+) cells, specifically (Figure 1d). These data show that the presence of DC-SIGN in KSHV-infected lymphocytes is highly variable. Finally, we analyzed the B cell subset distribution for DC-SIGN+ B cells in Mock and KSHV-infected conditions and found no significant influence of infection on DC-SIGN distribution (Figure 1e). Taken together, these data show minimal impact of KSHV infection on DC-SIGN expression and distribution, and indicate that expression of DC-SIGN within infected cells varies substantially based on donor. Thus, these results do not strongly support or entirely refute the hypothesis that DC-SIGN is used as an attachment receptor for KSHV infection in B lymphocytes. 

### 3.3. Neutralization of DC-SIGN Increases KSHV Infection of B Lymphocytes

**Figure 2:**
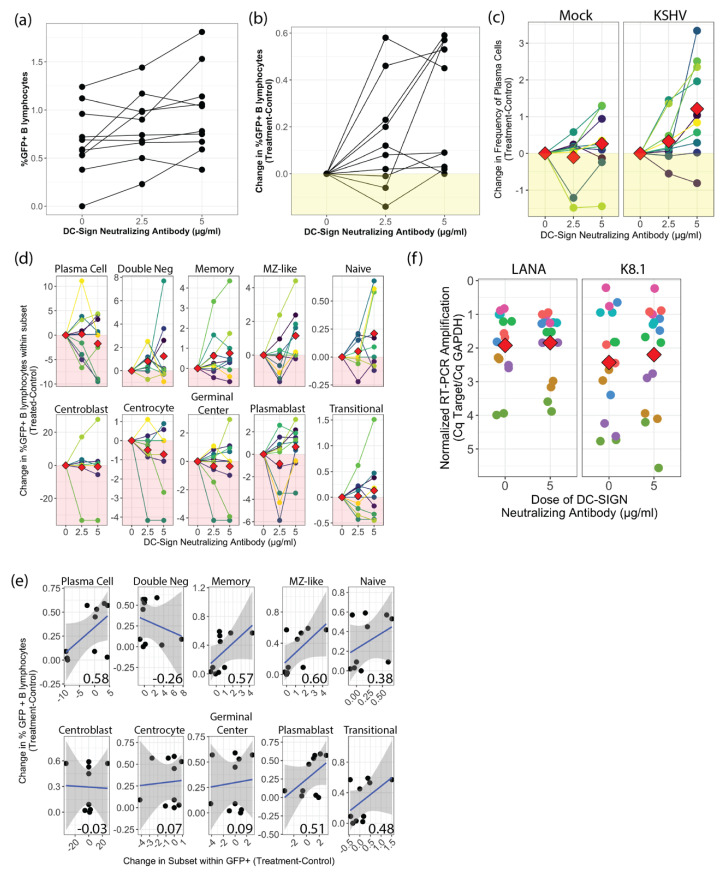
Effect of DC-SIGN neutralization on KSHV infection in tonsil B lymphocytes. (**a**) Flow cytometry analysis of KSHV infection of 10 unique tonsil specimens based on GFP+ B lymphocytes at 3 dpi with indicated doses of DC-SIGN neutralizing antibody (n.s.) (**b**) analysis as in (a) normalized within samples to the 0µg/mL dose in order to remove between-sample variability. *p* = 0.02 comparing 0 µg/mL to 5 µg/mL using Student’s T-test with Holm correction for multiple comparisons. (**c**) Flow cytometry analysis of plasma cell frequencies at 3dpi with indicated doses of DC-SIGN neutralizing antibody in Mock and KSHV-infected conditions. Two-way repeated measures ANOVA analysis reveals a statistically significant influence of mAb treatment on PC frequency (*p* = 0.003, F = 8.3) (**d**) normalized analysis of subset targeting by KSHV (treatment-control) for indicated doses of DC-SIGN neutralizing antibody (n.s.) (**e**) Normalized correlation analysis showing linear relationships (Pearson’s correlation coefficient) comparing the change in frequency of GFP within B cell subsets and the change in overall GFP frequency at 5 µg/mL neutralizing antibody dose. Power analysis indicates with this sample number correlations with r ≥ 0.77 are statistically significant. (**f**) replicate infections as in (a) with or without DC-SIGN neutralizing antibody at 5 µg/mL were harvested into trizol at 3 dpi and RT-PCR analysis was performed for GAPDH (human housekeeping gene), LANA (viral latent) and K8.1 (viral lytic) transcripts. No-RT control reactions were used to validate that DNA was not the source of amplification. Viral target Cq values were normalized to internal GAPDH controls and red diamonds indicate means of 8 unique tonsil specimens with two technical replicates per target. (n.s.).

In order to more directly determine whether DC-SIGN is required for KSHV entry into B lymphocytes, we performed experiments in which we blocked DC-SIGN using increasing concentrations of a DC-SIGN neutralizing antibody prior to infection and assessed the magnitude and distribution of KSHV infection at 3 dpi. Surprisingly, these results revealed increased KSHV infection at 3 dpi in most samples (Figure 2a). In order to account for the variable susceptibility of tonsil samples to KSHV infection in our model [5], we analyzed these data as a change in GFP using controls with no antibody treatment as a normalization factor. In this analysis we can clearly see that the majority of tonsils included in this analysis show increased infection in response to DC-SIGN neutralization, and the difference was statistically significant (*p* = 0.02) at the 5 µg/mL dose (Figure 2b). 

In order to determine whether the increase in GFP+ B cells with DC-SIGN neutralization was due to increased frequency or targeting of a specific B cell subtype, we performed subset analysis on these samples. Analysis of overall subset frequencies (infected and uninfected) in these cultures at 3 dpi revealed that most subsets did not change with DC-SIGN neutralization in either the Mock or KSHV-infected conditions (Figure S2). However, we did observe a statistically significant increase in the CD138+ plasma cell population at the 5 µg/mL dose only in the KSHV-infected conditions (Figure 2c). This observation is particularly interesting given our recent publication showing that plasma cells are highly targeted by KSHV early in infection [5]. 

Next, we wanted to determine whether neutralization of DC-SIGN influences the distribution of KSHV infection within B cell subsets. In these experiments, if a particular subset shows decreased targeting by KSHV in the presence of DC-SIGN neutralization, we can conclude that DC-SIGN is important for viral entry in that cell type. Our results show no statistically significant increase or decrease in KSHV infection of any specific B cell subset. However, infection of memory, MZ-like and naive B cells was increased in most of the samples (Figure 2d). These results, therefore, do not identify any B cell subset in which DC-SIGN is critical for entry. 

We next wanted to determine whether increased the overall infection we observed with DC-SIGN neutralization (Figure 2b) was correlated with changes in infection of any B cell subsets. In order to examine this, we plotted the change in frequency of GFP within B cell subsets against overall change in GFP frequency at the 5µg/mL neutralizing antibody dose and calculated correlation coefficients via the Pearson method (Figure 2e). Although power analysis indicates that these correlations are not strong enough to be statistically significant with this sample number, we observe that samples with increased GFP also displayed increased proportions of infected plasma, MZ-like and memory B cell subsets. These observations indicate that these particular subsets do not depend upon DC-SIGN for KSHV entry. We hypothesized that DC-SIGN neutralization was increasing overall infection by increasing targeting of B cell subsets that undergo lytic replication, thereby acting as a point of expansion for the virus. To test this hypothesis, we performed RT-PCR analysis for LANA (a latent gene) and K8.1 (a lytic gene) on eight unique tonsil specimens with and without DC-SIGN neutralization at 5 µg/mL. These results show that there is no difference in lytic gene expression between untreated cultures and cultures treated withDC-SIGN neutralizing antibodies (Figure 2f). These results suggest that the mechanism for increased infection with DC-SIGN neutralization is not increased viral spread within the cultures.

### 3.4. KSHV gH Is not Required for KSHV Infection of Tonsil Derived B Lymphocytes

**Figure 3:**
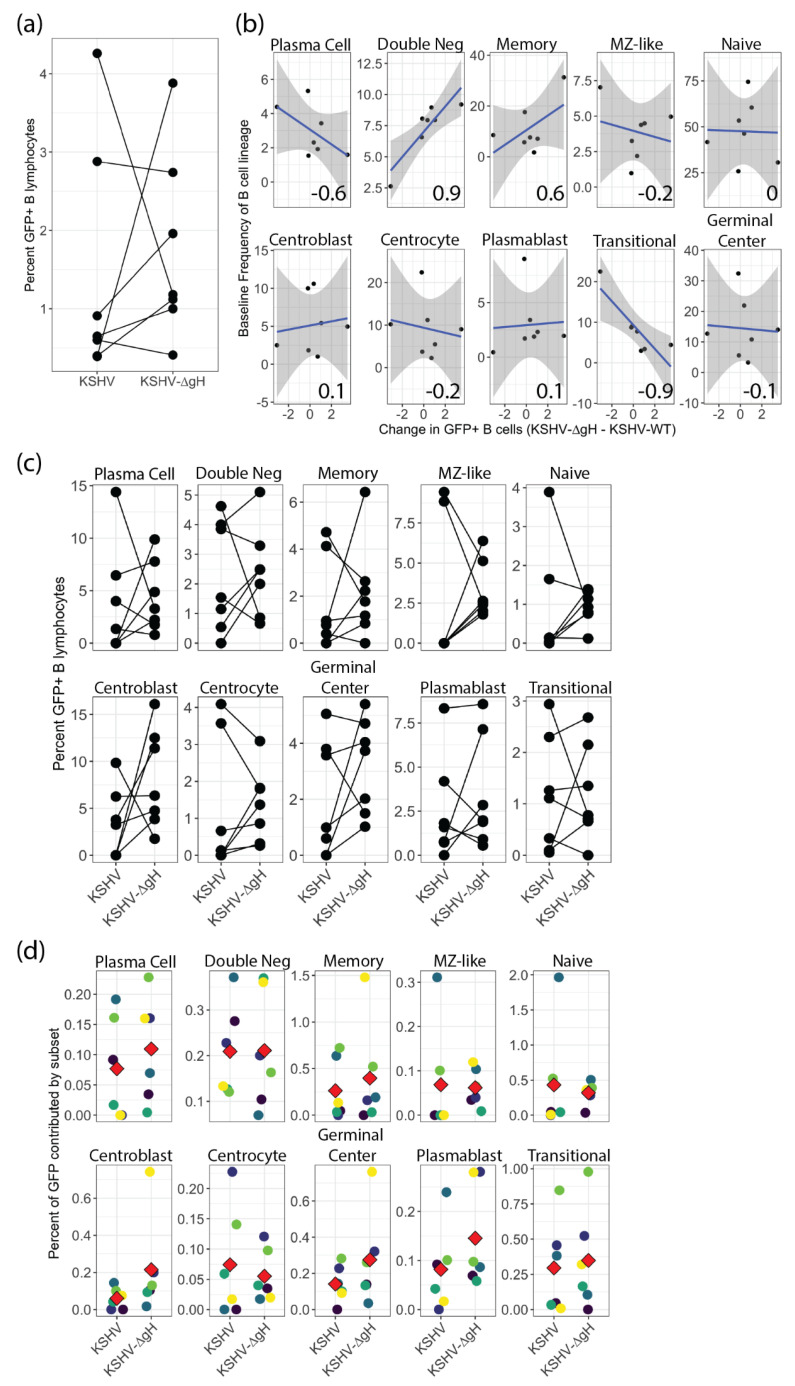
KSHV-∆gH infection in tonsil B lymphocytes. (**a**) Flow cytometry analysis of 7 unique tonsil specimens infected with KSHV-WT and KSHV-∆gH based on GFP+ B lymphocytes at 3 dpi (**b**) Correlation analysis showing linear relationships (Pearson’s correlation coefficient) between baseline frequency of B-cell subsets and the change in GFP between KSHV-∆gH and KSHV-WT on a per-sample basis. Power analysis indicates with this sample number correlations with r ≥ 0.86 are statistically significant. (**c**) Distribution of KSHV-WT and KSHV-∆gH infected cells (GFP+) within B cell subsets at 3 dpi (n.s.) (**d**) Distribution of B-cell subsets within infected (GFP+) fraction for KSHV-WT and KSHV-∆gH. Red diamonds indicate mean for each condition (n.s.). Where indicated, data point color scheme indicates unique tonsil specimens and can be compared between sub-panels within each portion of the figure.

We recently showed that a mutant KSHV virus lacking gH, is globally defective for entry into adherent cell lines including epithelial, endothelial and fibroblast cell lines but was still able to infect the MC116 lymphoma cell line [6]. These results suggest that gH may be dispensable for entry into B cells. In order to test this hypothesis in our primary tonsil lymphocyte system, we utilized the same mutant virus that was constructed for this previous study (KSHV-∆gH). We performed infections with 7 unique tonsil lymphocyte samples using KSHV-WT, KSHV-∆gH and Mock infection as described above, and assessed the distribution of KSHV infection at 3 dpi using flow cytometry. It should be noted that because KSHV-∆gH cannot infect fibroblasts we cannot use our normal system for functional titration of the virus stock to normalize infection. Thus, for these experiments we performed titration of the KSHV-WT stock as usual on fibroblasts to determine the optimal infectious dose, and then used genome quantitation of both KSHV-WT and KSHV-∆gH virus stocks in order to calculate an equivalent genome dose for KSHV-∆gH. These results reveal that KSHV-∆gH infects tonsil-derived B cells at levels comparable to those seen with KSHV-WT. Interestingly, four of seven tonsil samples showed higher levels of GFP+ B lymphocytes in KSHV-∆gH compared to WT (Figure 3a). We wanted to determine whether the difference in infection efficiency with KSHV-WT vs. KSHV-∆gH was related to the levels of B cell subsets in the tonsil samples at the time of infection. To do this analysis, we calculated the difference between the percent of GFP+ B cells with each virus on a per-sample basis (KSHV-∆gH minus KSHV-WT) and compared this value to the sample-specific baseline frequency of each B cell subset. These data reveal that KSHV-∆gH infectivity is highly correlated with baseline levels of double negative B cells (an atypical memory B cell subtype), but that KSHV-∆gH had lower infectivity in samples with high levels of transitional B cells (Figure 3b). These results may suggest that KSHV infection of transitional B cells relies more heavily upon gH-dependent entry mechanisms compared to entry into double negative B cells. When we examined the distribution of GFP within B cell subsets at 3 dpi for these experiments, we found that there was no significant difference in B cell targeting between KSHV-WT and KSHV-∆gH (Figure 3c). Finally, we wanted to determine whether the distribution of B cell subsets within the infected (GFP+) fraction was different between KSHV-WT and KSHV-∆gH. This analysis showed that there was also no significant difference between KSHV-WT and KSHV-∆gH infection (Figure 3d). Thus, neither the amount of infection within any B cell subset nor the proportion of B cell subsets within the infected fraction differed between KSHV-WT and KSHV-∆gH. Taken together, these results indicate that KSHV-∆gH (a virus which is globally defective in non-B cell types) can infect primary B cells, and displays similar infectivity and B cell subset tropism when compared to KSHV-WT. We would conclude from these data that overall susceptibility of individual tonsil specimens to infection with KSHV-∆gH is dependent upon the B cell composition of the specimen, suggesting that gH does play a role in entry into B lymphocytes. However, gH is not strictly required for KSHV entry into any B cell subset, suggesting that this glycoprotein does not play a pivotal role in KSHV infection of B lymphocytes in human tonsil. 

### 3.5. Depletion of DC-SIGN in KSHV-WT and KSHV-∆gH Infections Reveals Multiple, Entry Mechanisms for KSHV in Tonsil B Lymphocytes

**Figure 4:**
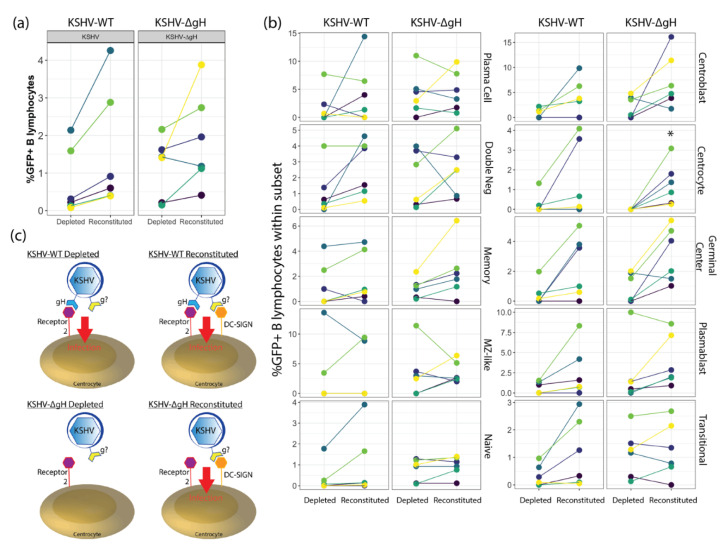
Effect of DC-SIGN Depletion on KSHV-WT and KSHV-∆gH infection in tonsil B lymphocytes. (**a**) Flow cytometry analysis of 6 unique tonsil specimens in which DC-SIGN+ cells were depleted, or depleted fractions were reconstituted with isolated DC-SIGN+ B cells prior to KSHV infection with KSHV-WT or KSHV-∆gH and analyzed 3 days post infection (n.s.) (**b**) analysis as in (**a**) showing distribution of infected (GFP+) cells within B-cell subsets. * *p* = 0.03 for depleted vs. reconstituted within KSHV-∆gH infection of centrocytes (**c**) Model for entry mechanisms in the presence and absence of DC-SIGN and/or ∆gH for centrocytes.

In order to examine whether KSHV-∆gH interacts with DC-SIGN to enter into B lymphocytes and to validate our earlier DC-SIGN neutralization data (Figure 2) via another method, we performed experiments with 6 unique tonsil specimens where we isolated total B lymphocytes and performed a second step where we separated DC-SIGN+ and DC-SIGN- B cells. Because DC-SIGN+ cells represent a minor population in tonsil B cells (Figure 1a) we did not have sufficient cell numbers to infect the populations separately. Therefore, we adopted a strategy where we either infected DC-SIGN-depleted populations or reconstituted the depleted population with DC-SIGN+ B cells prior to infection. Analysis of KSHV infection in these cultures revealed that KSHV-WT infection is uniformly higher in DC-SIGN reconstituted cultures compared to DC-SIGN depleted cultures. This trend was similar in KSHV-∆gH infected cultures with the exception of one tonsil sample in which infection was increased in reconstituted culture for KSHV-WT and decreased in the same condition with KSHV-∆gH (Figure 4a). Next, we examined the distribution of GFP within B cell subsets for DC-SIGN depleted vs. reconstituted cultures in order to determine whether DC-SIGN is required for entry into any particular B cell subset. Consistent with our neutralization data, depletion of DC-SIGN did not abolish infection of any B cell subsets with KSHV-WT. Consistent with our previous analysis (Figure 3) there was no statistically significant difference in infection comparing WT to ∆gH for either depleted or reconstituted fractions. For KSHV-WT, although infection of most subsets tended to be higher in the reconstituted fractions, there was no significant difference between DC-SIGN depleted and reconstituted cultures for any subset with KSHV-WT. Results were similar for KSHV-∆gH with the exception of centrocytes (the CD77 negative sub-population of germinal center B cells) where infection was absent in the DC-SIGN depleted culture vs. the DC-SIGN reconstituted culture (*p* = 0.03) (Figure 4b). These results support our previous conclusions that DC-SIGN plays a minor role as a receptor for KSHV entry into tonsil-derived B cells, and that the KSHV glycoprotein gH is not required for entry into any B cell subsets. However, our data reveal that in the absence of both the DC-SIGN receptor and the gH glycoprotein, KSHV cannot infect centrocytes. This implies that KSHV has only two entry mechanisms for centrocytes: (1) a gH-dependent mechanism that uses a receptor other than DC-SIGN, and (2) a gH-independent mechanism that uses DC-SIGN as the receptor. In KSHV-WT, both entry mechanisms are functional and no difference is seen when DC-SIGN is depleted because the gH-dependent entry mechanism is intact. When gH is absent but DC-SIGN is present (KSHV-∆gH reconstituted) entry into centrocytes is mediated by the interaction of another KSHV-gp and DC-SIGN. When gH is absent and DC-SIGN is absent, KSHV has no entry mechanism for centrocytes (Figure 4c). 

Taken together, our examination of the roles of KSHV-gH and cellular DC-SIGN reveals that:

1. KSHV-gH is not required for entry into tonsil-derived B lymphocytes.
2. KSHV-gH can participate in KSHV entry into centrocytes via a DC-SIGN independent entry mechanism.
3. DC-SIGN is not necessary for KSHV entry into any B cell subset.
4. DC-SIGN does play a role in KSHV entry into tonsil-derived B cells, but in all B-cell subtypes alternative entry mechanisms exist.

Our results presented in this study provide a first glimpse into the complexity of KSHV entry in the lymphocyte compartment and highlight that multiple subset-dependent entry mechanisms are employed by KSHV which depend upon multiple cellular receptors and multiple KSHV glycoproteins. Further studies are needed to establish the roles of additional receptor-gp interactions in these important cell types in order to rationally design therapies and vaccine strategies that will effectively limit KSHV entry into, and spread within, the human immune system.

## 4. Discussion

Our results presented in this study indicate that KSHV can use DC-SIGN as an attachment/entry factor, but DC-SIGN is not required for entry into any B cell subsets. In particular, depletion of DC-SIGN-expressing B cells had a more profound effect than antibody-mediated neutralization of DC-SIGN. Indeed, KSHV infection was overall increased in our neutralization experiments. These results contrast significantly with the only previous study to perform an in-depth analysis on the role of DC-SIGN as an entry receptor for KSHV, which was performed by Rappocciolo et al. in 2008 [8]. This particular study utilized both unstimulated tonsil-derived B cells and peripheral blood B-cells that were activated with CD40 ligand (CD40L) and interleukin 4 (IL-4) as target cells and wild-type KSHV virions derived from BCBL-1 PEL cells as virus inoculum. In our results, we show a lower percentage of DC-SIGN+ B cells compared to this previous study possibly indicating that our tonsil B cells are less activated. One reason for this discrepancy may be methodological. We avoid using Ficoll-Hypaque and similar agents for lymphocyte purification and we utilize negative selection strategies for B cell isolation in order to avoid aberrant activation of cells in our system. Importantly, we utilized multi-color flow cytometry for our analysis method which allows us to see KSHV infection using the constitutively expressed GFP reporter present in the BAC16 genome (which we have previously shown is a reliable marker for KSHV tropism [5]) alongside surface antigen markers for B cell subsets. These techniques were distinct from the previous study which analyzed B cells as a single population and utilized virus detection methods that were biased towards lytic replication as opposed to quantitating infected cell numbers.

When we employed a neutralizing antibody to disrupt any interaction between DC-SIGN and KSHV glycoproteins, we observed significantly increased KSHV infection at the highest dose (5 µg/mL), which was also the optimal dose for inhibition of DC-SIGN - ICAM3 interaction during characterization of the neutralizing antibody by the manufacturer. These results differ from those reported in Rappocciolo et al., where use of the same anti-DC-SIGN neutralizing antibody clone but at much higher concentrations (20 µg/mL) effectively blocked KSHV infection of activated peripheral B-cells [8]. Aside from the differing mAb concentrations, our culture model recapitulates the total tonsil lymphocyte environment after infection. Thus, we might hypothesize this mAb enhancement effect may be mediated by activation of DC-SIGN signaling altering other attachment factors or via altered interactions involving non-B cells in the culture model. Our analysis of the distribution of KSHV infection within B-cell subsets did not reveal any change in KSHV targeting of B-cells with DC-SIGN neutralization, and RT-PCR results show no increase in lytic replication in culture where the neutralizing antibody was used. Thus, our data provide no evidence that the increase in overall infection with DC-SIGN neutralization is a result of altered KSHV targeting or spread. We did observe an increase in total plasma cell numbers in DC-SIGN neutralized, KSHV-infected lymphocyte cultures. Our previous work has shown that our lymphocyte culture system does not favor the survival of plasma cells, but KSHV infection increases overall plasma cell numbers at 3 dpi [5]. Recently, activation of DC-SIGN signaling via antibody binding was shown to promote survival of B lymphoma cells [18]. Thus, the combination of DC-SIGN neutralization and KSHV infection may synergistically promote the survival of plasma cells in our culture system, and we speculate that the presence of plasma cells promotes KSHV infection via unknown mechanisms that are possibly related to the cytokine milieu. Overall, our results collectively show that DC-SIGN is not required for KSHV entry into any B-cell subset.

To date, it is unknown which viral glycoproteins are essential for KSHV entry in tonsil derived B lymphocytes, and this gap in our understanding limits the rational design of vaccines designed to limit the ability of KSHV to invade the immune system. We recently showed that gH is essential for KSHV entry into epithelial cells, endothelial cells and fibroblasts but is not required for entry into the MC116 B-cell lymphoma cell line [6]. Our current study extends these findings and shows that gH is also not required for entry into primary tonsil lymphocytes. Interestingly, our results revealed tonsil donor-specific differences in the infectivity of KSHV-∆gH compared to KSHV-WT, and that these differences are correlated to the frequencies of transitional and double negative B cells in the original tonsil sample. These results provide a critical insight indicating that vaccination strategies concentrated on KSHV-gH may leave the immune system vulnerable to infection. However, our DC-SIGN depletion experiments show that there is a gH-dependent mechanism for KSHV entry into centrocytes. Thus, although gH is not strictly required for entry into any B cell type, gH-dependent entry mechanisms do exist for tonsil-derived B cells. Taken together, our results indicate that KSHV entry in B lymphocytes is complex, multi-factoral and that subset-dependent entry mechanisms exist but, in all cases, KSHV has multiple entry points to exploit on any given B cell subset.

Our study does have some limitations that require further research. In particular, for the neutralization experiment we chose to utilize a neutralizing antibody that may not be optimal for the inhibition of DC-SIGN and KSHV glycoprotein interactions. The anti-DC-SIGN antibody we utilized is optimized to neutralize the interaction of DC-SIGN with ICAM-3/CD50. It should be noted that the DC-SIGN receptor is a tetramer protein composed of four individual domains: the C-terminal carbohydrate recognition domain (CRD), the neck-repeat region, the transmembrane domain, and the N terminal cytoplasmic tail. Due to DC-SIGN’s role in the process of viral infection and its structural complexity, many groups believe that blocking the sugar binding site, ICAM-3 epitope in the carbohydrate recognition domain (CRD) will overall inhibit DC-SIGN and not allow the virus to enter. This assumption is believed to be true because the ICAM-3 epitope is located in the center of the carbohydrate recognition domain where most molecular processes are mediated in the DC-SIGN receptor [19]. Importantly, the biochemistry of any interactions between DC-SIGN and KSHV glycoproteins has not been established. The fact that our results show increased infection with DC-SIGN neutralization could imply that the neutralizing antibody may be binding to a different epitope on DC-SIGN and that the antibody is not neutralizing the actual interaction of the viral glycoproteins to DC-SIGN. More research is needed to decipher the real interactions between DC-SIGN and KSHV glycoproteins to be able to fully neutralize those specific interactions. Moreover, our results with DC-SIGN neutralization highlight the need to better establish the role of DC-SIGN signaling in our tonsil lymphocyte cultures and its impact on KSHV infection.

Future studies are also needed to determine if the other conserved glycoproteins: gB, gM, gN, ORF4 and gpK8.1 are essential for KSHV entry in tonsil lymphocytes. Through such studies, we will have the opportunity to unravel different entry mechanisms for KSHV in tonsil derived B cells and to determine what viral glycoproteins are essential for entry. In addition, future studies are needed to determine the specific cellular receptors that KSHV uses to enter each susceptible B cell subset and how these receptors change based on inflammatory and metabolic factors that may influence the spread of KSHV within a human host. Previous studies have shown that cellular receptors for KSHV can be modulated by metabolic factors such as high glucose associated with diabetes [20]. Moreover, KSHV replication has been shown to modify metabolic parameters such as lipogenesis [21], creating a positive feedback loop between viral spread, persistence and pathogenesis. Understanding the fundamental virology of KSHV entry in the lymphocyte compartment will be essential to rationally designing treatment strategies that limit KSHV spread and pathogenesis within a human host. Moreover, the rational design of vaccines to prevent the person-to-person spread of KSHV, and thereby limit the public health impact of KSHV-associated malignancies, will depend upon our understanding of how KSHV invades the immunological compartment. The results presented herein represent a critical first step in filling this critical gap in our understanding of KSHV virology.

## Figures and Tables

**Table 1:** Primers and probes for the RT-PCR assays used in the study.

Target	Outer Fwd	Outer Rev	Inner Fwd	Inner Rev	Probe	Amplicon Size (bp)
GAPDH	5′-TCGGAGTCAACGGATTTGGT-3′	5′-GGGTCTTACTCCTTGGAGGC-3′	5′-TCGGAGTCAACGGATTTGGT-3′	5′-GGGTCTTACTCCTTGGAGGC-3′	5′[HEX]-ACGCCACAGTTTCCCGGAGG-[BHQ1]3′	92
LANA	5′-AATGGGAGCCACCGGTAAAG-3′	5′-CGCCCTTAACGAGAGGAAGT-3′	5′-AATGGGAGCCACCGGTAAAG-3′	5′-CGCCCTTAACGAGAGGAAGT-3′	5’[6FAM]-ACACAAATGCTGGCAGCCCG-[BHQ1]3′	77
K8.1	5′- ACCGTCGGTGTGTAGGGATA-3′	5′-TCGTGGAACGCACAGGTAAA-3′	5′-ACCGTCGGTGTGTAGGGATA-3′	5′-TCGTGGAACGCACAGGTAAA-3′	5′[Cy5]-TGCGCGTCTCTTCCTCTAGTCGTTG-[BHQ1]3′	87

**Table 2:** Subset definitions for B cell subsets used in this study.

Subset	Molecular Markers
Plasma	CD19^+^, CD20^+/−^, CD138^+(Mid to High)^, CD38^−^
Transitional	CD19^+^, CD138^−^, CD38^Mid^, IgD^+ (Mid to High)^
Plasmablast	CD19^+^, CD138^−^, CD38^High^, IgD^+/− (mostly-)^
Germinal Center	CD19^+^, CD138^−^, CD38^Mid^, IgD^−^
GC-Centrocytes	CD19^+^, CD138^−^, CD38^Mid^, IgD^−^, CD77^−^
GC-Centroblasts	CD19^+^, CD138^−^, CD38^Mid^, IgD^−^, CD77^+^
Naïve	CD19^+^, CD138^−^, CD38^Low^, CD27^−^, IgD^+ (Mid to High)^
Marginal Zone Like (MZ-Like)	CD19^+^, CD138^−^, CD38^Low^, CD27^+ (Mid to High)^, IgD^+ (Mid to High)^
Memory	CD19^+^, CD138^−^, CD38^Low^, CD27^+ (Mid to High)^, IgD^−^
Double Negative	CD19^+^, CD138^−^, CD38^Low^, CD27^−^, IgD^−^

## Data Availability

Raw data files from this study are available from the corresponding author upon request.

## Supplementary Materials

The following are available online at https://www.mdpi.com/article/10.3390/v13081512/s1, Figure S1: Gating scheme for tonsil B lymphocyte subset immunophenotyping, Figure S2: Effect of DC-SIGN neutralization on lymphocyte subset frequencies at 3 dpi.
