# Supplementary material for: Suppression of DC-SIGN and gH Reveals Complex, Subset-Specific Mechanisms for KSHV Entry in Primary B Lymphocytes
Source: Viruses. 2021 Jul 31;13(8):1512. doi: 10.3390/v13081512 (PMC8402705; doi:10.3390/v13081512)
Supplement: Supplementary file 1 [file viruses-13-01512-s001.zip › viruses-1299312-supplementary.pdf]

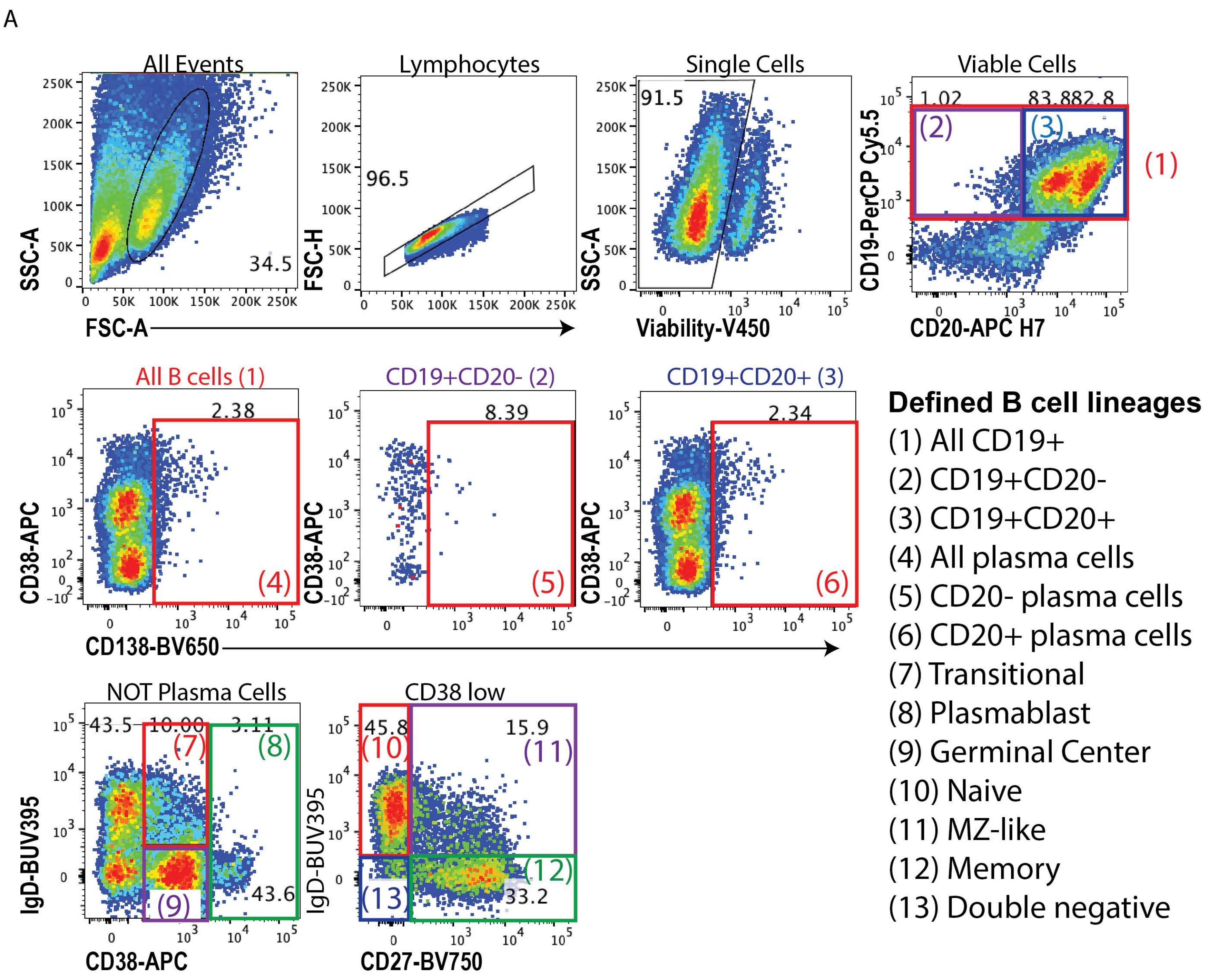

Supplemental Figure S1: Sample gating scheme for the B cell lineages in this study

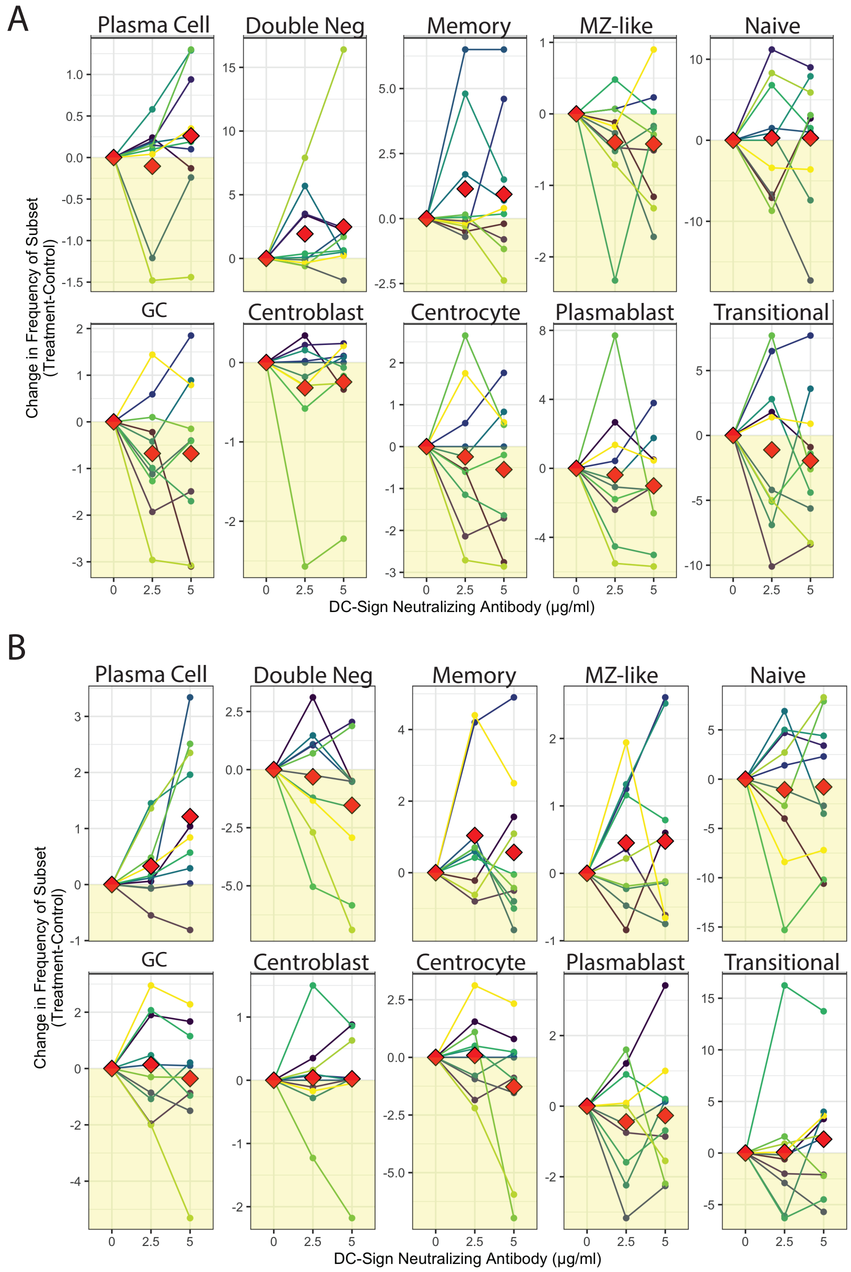

Supplemental Figure S2: Changes in lineage frequencies (Treatment-Control) with DC-SIGN neutralization at 3dpi for (A) Mock infected and (B) KSHV-WT infected cultures. Red diamonds indicate the mean of all tonsil samples for the specific lineage/condition.
